# *ERCC6L*, a DNA helicase, is involved in cell proliferation and associated with survival and progress in breast and kidney cancers

**DOI:** 10.18632/oncotarget.14998

**Published:** 2017-02-02

**Authors:** Shao-Yan Pu, Qin Yu, Huan Wu, Jian-Jun Jiang, Xiao-Qiong Chen, Yong-Han He, Qing-Peng Kong

**Affiliations:** ^1^ State Key Laboratory of Genetic Resources and Evolution, Kunming Institute of Zoology, The Chinese Academy of Sciences, Kunming 650223, China; ^2^ KIZ/CUHK Joint Laboratory of Bioresources and Molecular Research in Common Diseases, Kunming 650223, China; ^3^ Kunming College of Life Science, University of Chinese Academy of Sciences, Beijing 100049, China

**Keywords:** excision repair cross-complementation group 6 like, cancer, proliferation, MAPK

## Abstract

By analyzing 4987 cancer transcriptomes from The Cancer Genome Atlas (TCGA), we identified that excision repair cross-complementation group 6 like (ERCC6L), a newly discovered DNA helicase, is highly expressed in 12 solid cancers. However, its role and mechanism in tumorigenesis are largely unknown. In this study, we found that *ERCC6L* silencing by small interring RNA (siRNA) or short hairpin RNA (shRNA) significantly inhibited the proliferation of breast (MCF-7, MDA-MB-231) and kidney cancer cells (786-0). Furthermore, *ERCC6L* silencing induced cell cycle arrest at G0/G1 phase without affecting apoptosis. We then performed RNA sequencing (RNA-seq) analysis after *ERCC6L* silencing and identified that RAB31 was markedly downregulated at both the transcriptional and translational levels. Its downstream protein, phosphorylated MAPK and CDK2 were also inhibited by *ERCC6L* silencing. The xenograft experiment showed that silencing of *ERCC6L* strikingly inhibited tumor growth from the 7th day after xenograft in nude mice. In addition, higher *ERCC6L* expression was found to be significantly associated with worse clinical survival in breast and kidney cancers. In conclusion, our results suggest that *ERCC6L* may stimulates cancer cell proliferation by promoting cell cycle through a way of RAB31-MAPK-CDK2, and it could be a potential biomarker for cancer prognosis and target for cancer treatment.

## INTRODUCTION

The rapid accumulation of cancer transcriptomic data provides a good opportunity for us to identify new cancer related genes and to deeply understand the molecular mechanism of the disease. We analyzed a total of 4987 transcriptomes of 12 cancer types from The Cancer Genome Atlas (TCGA) and found that excision repair cross-complementation group 6-like (*ERCC6L*) was highly expressed in almost all cancer types. *ERCC6L* is a newly discovered DNA helicase, also called PICH (Polo-like kinase 1-interacting checkpoint “helicase”). In 2005, Xu et al. first cloned *Ercc6l* from mouse embryos with an aim of looking for embryonic development related proteins [1]. Later, some studies reported that *ERCC6L* was overexpressed in embryonic heart, brain and other tissues [1]. Li et al. also showed that *ERCC6L* expression was significantly higher in the youngest deer than other age groups [2]. These studies indicate that *ERCC6L* is critical to embryonic development.

In 2007, Baumann et al. revealed that *ERCC6L* could combine with a mitosis regulation kinase (Polo-like kinase 1, *PLK1*) and was involved in remodeling centromeric chromatin [3]. *PLK1* plays wide roles in regulating cell division, cell proliferation and other biological processes, and is considered as a genetic marker in the development of tumors [4, 5]. The high expression of *ERCC6L* plus its role in embryonic development as well as the involvement of remodeling centromeric chromatin promote us to hypothesize that it may play a role in tumorigenesis. In fact, *ERCC6L* was showed to be required to affect mitotic centromere and chromosome structure in mammal cells [3, 6, 7]. For example, Hubner et al. found that the functional impairment of *ERCC6L* increased human embryonic kidney cell division by siRNA [8]. Rouzeau et al. also reported that *ERCC6L* can increase centromere division in the late fibroblast cell division [9]. However, the role of highly expressed *ERCC6L* in tumors has never been investigated.

In this study, we took breast and kidney cancers for examples and demonstrated for the first time that *ERCC6L* silencing inhibited the proliferation of cancer cells, and tumor growth in nude mice. We also reported significant associations of *ERCC6L* expression with clinical survival and progress in cancers. These results altogether suggest *ERCC6L* to be a new target for cancer treatment.

## RESULTS

### *ERCC6L* was significantly overexpressed in various cancers

**Figure 1:**
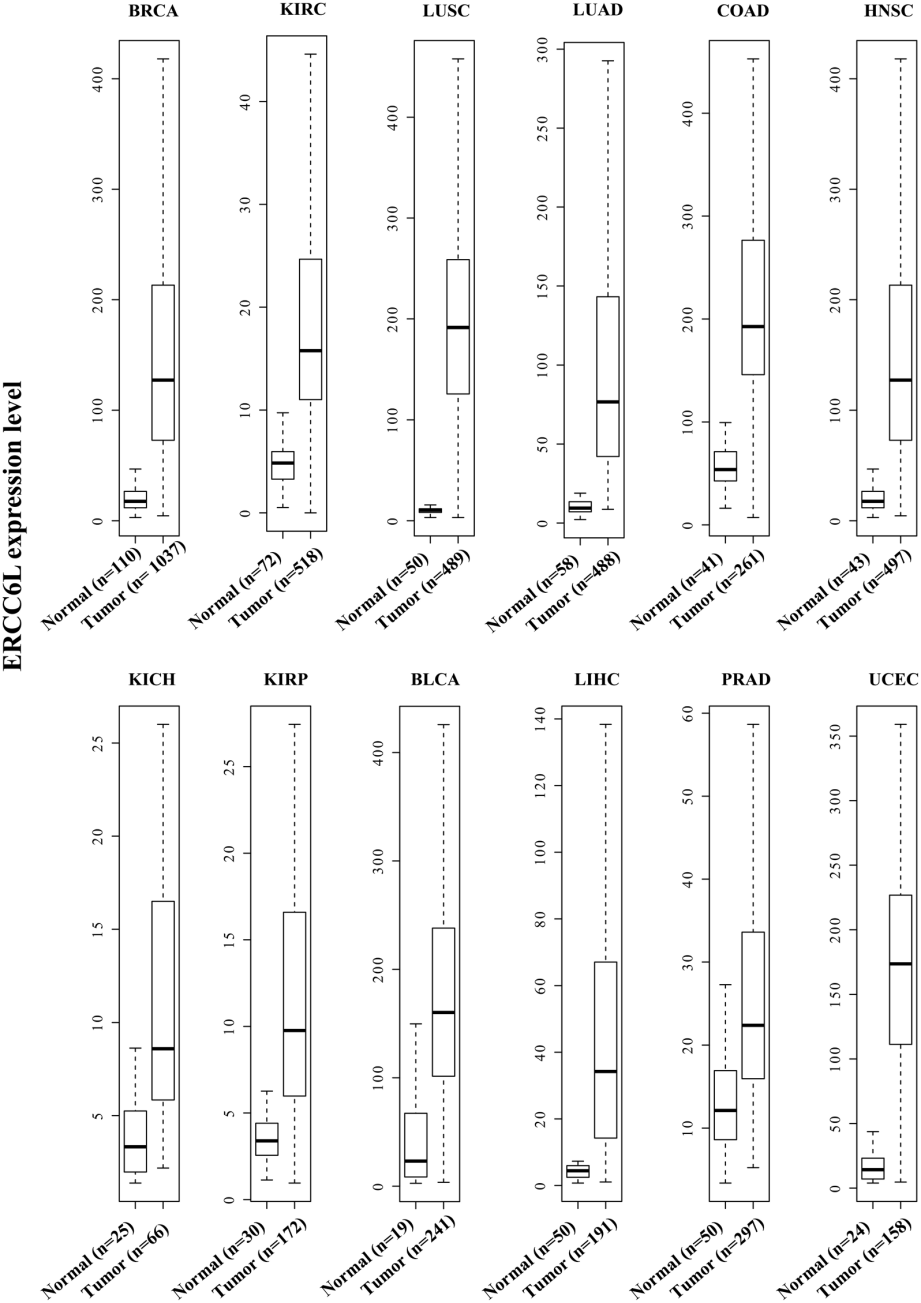
*ERCC6L* expression in 12 cancer types from The Cancer Genome Atlas (TCGA) All *p* values are less then 4.5E-11 except for KICH (p = 0.0003).

We analyzed 4987 transcriptome of 12 cancer types from The Cancer Genome Atlas (TCGA) and found that *ERCC6L* was consistently overexpressed in all of the 12 cancers compared to their corresponding normal controls (all p<0.001, Figure 1). The results hint that abnormal expression of *ERCC6L* gene may play a role in tumorigenesis. As the sample size of breast and kidney cancers rank 1^st^ and 2^nd^ among the 12 cancer types (breast cancer: 1037 tumor vs. 110 normal tissues; kidney cancer: 518 tumor vs. 72 normal tissues) in TCGA, so we chose them as representatives to carry out the followed experiments.

### *ERCC6L* silencing inhibited proliferation and induced cell cycle arrest

**Figure 2:**
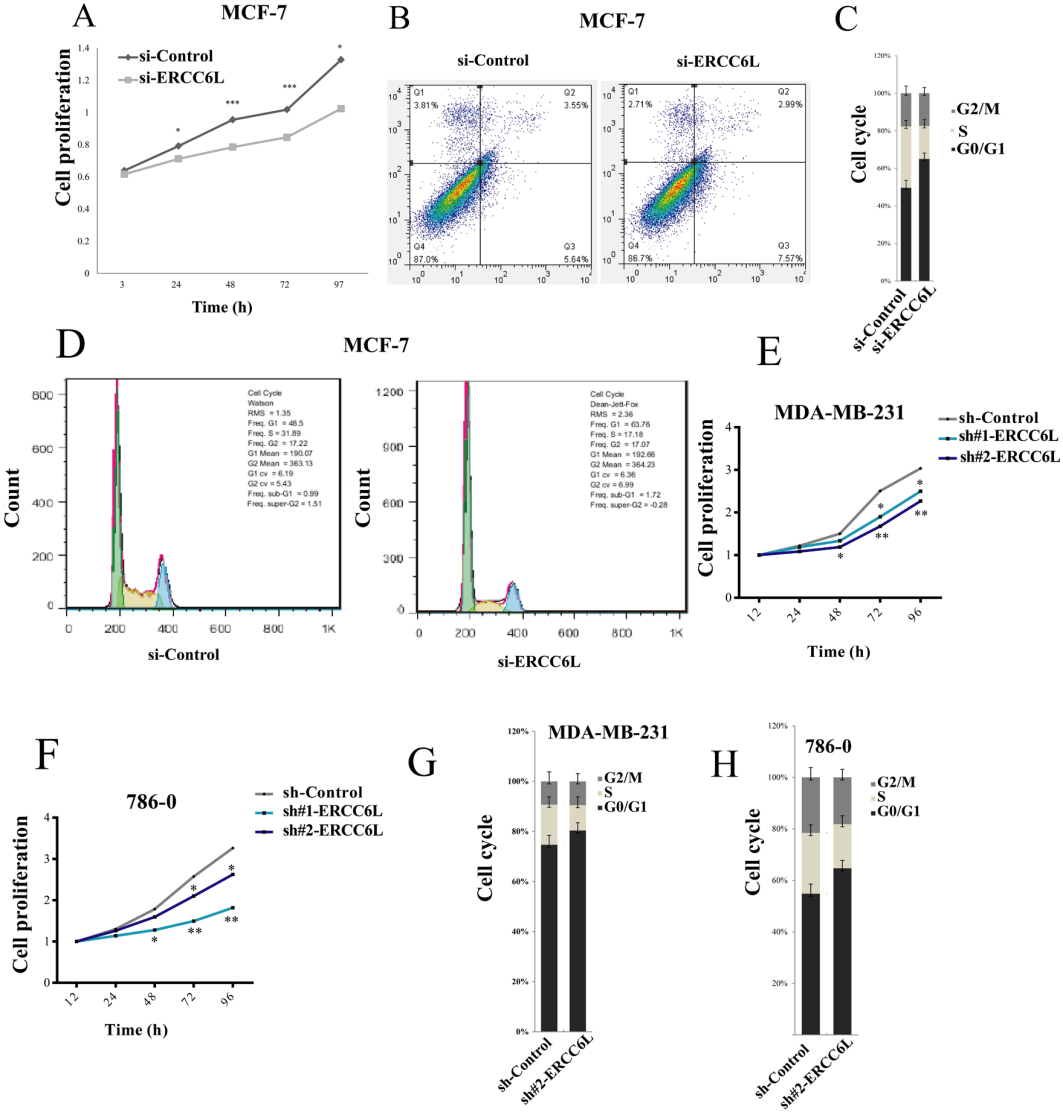
Effect of of *ERCC6L* knockdown on cell proliferation, apoptosis and cell cycle **A**. Effect of *ERCC6L* knockdown on cell proliferation at different time points in MCF-7 cells. **B**. Effect of of *ERCC6L* knockdown on cell apoptosis in MCF-7 cells. MCF7 cells were stained with Annexin V. **C-D**. Effect of *ERCC6L* knockdown on cell cycle in MCF-7 cells. Effect of *ERCC6L* knockdown on cell cycle significantly arrested at G0/G1 phase compared with the control in MCF-7 cells. The vertical axis shows the percentage of cells at different phases of cell cycle. **E**. Effect of *ERCC6L* knockdown on cell proliferation at different time points in MDA-MB-231 cell lines. **F**. Effect of *ERCC6L* knockdown on cell proliferation at different time points in 786-0 cells. **G**. Effect of *ERCC6L* knockdown on cell cycle in MDA-MB-231 cells. **H**. Effect of *ERCC6L* knockdown on cell cycle in 786-0 cells. *ERCC6L* were silenced by siRNA in MCF-7 cells, and were silenced by two different shRNAs (sh#1-ERCC6L and sh#2-ERCC6L) in both MDA-MB-231 and 786-0 cell lines. Error bars represent mean ± SD. *p<0.05 compared to the control group. **p<0.01 compared to the control group. ***p<0.001 compared to the control group.

We knocked down *ERCC6L* by small interfering RNA (siRNA) (Supplementary Figure 1A) and found that *ERCC6L* silencing (si-ERCC6L) significantly inhibited the proliferation of MCF-7 cells in 24, 48, 72 and 97 h (p<0.05, Figure 2A). We then detected cell apoptosis using Annexin V in si-ERCC6L and si-Control cells, but did not observe any differences between the two groups (Figure 2B). Next we detected the effect of *ERCC6L* inhibition on cell cycle in MCF-7 cells and found that *ERCC6L* silencing induced cell cycle arrest at G0/G1 phase compared to the control group (Figure 2C and 2D).

To verify the effect of *ERCC6L* on cell proliferation, we knocked down *ERCC6L* by shRNA (Supplementary Figure 1B) in another two cancer cell lines, a breast cancer cell line MDA-MB-231 and a kidney cancer cell line 786-0. The results showed that *ERCC6L* silencing by shRNA can also significantly inhibited the proliferation of MDA-MB-231 and 786-0 cells in 48, 72 and 96 h (p<0.05, Figure 2E and 2F). Similarly, *ERCC6L* silencing induced cell cycle arrest at G0/G1 phase compared to the control group in MDA-MB-231 and 786-0 cells (Figure 2G and 2H). These results indicate that the *ERCC6L* silencing was able to inhibit cell proliferation through inducing cell cycle arrest in multiple cancer cell lines.

### *ERCC6L* silencing inhibited RAB31 and MAPK expression

**Figure 3:**
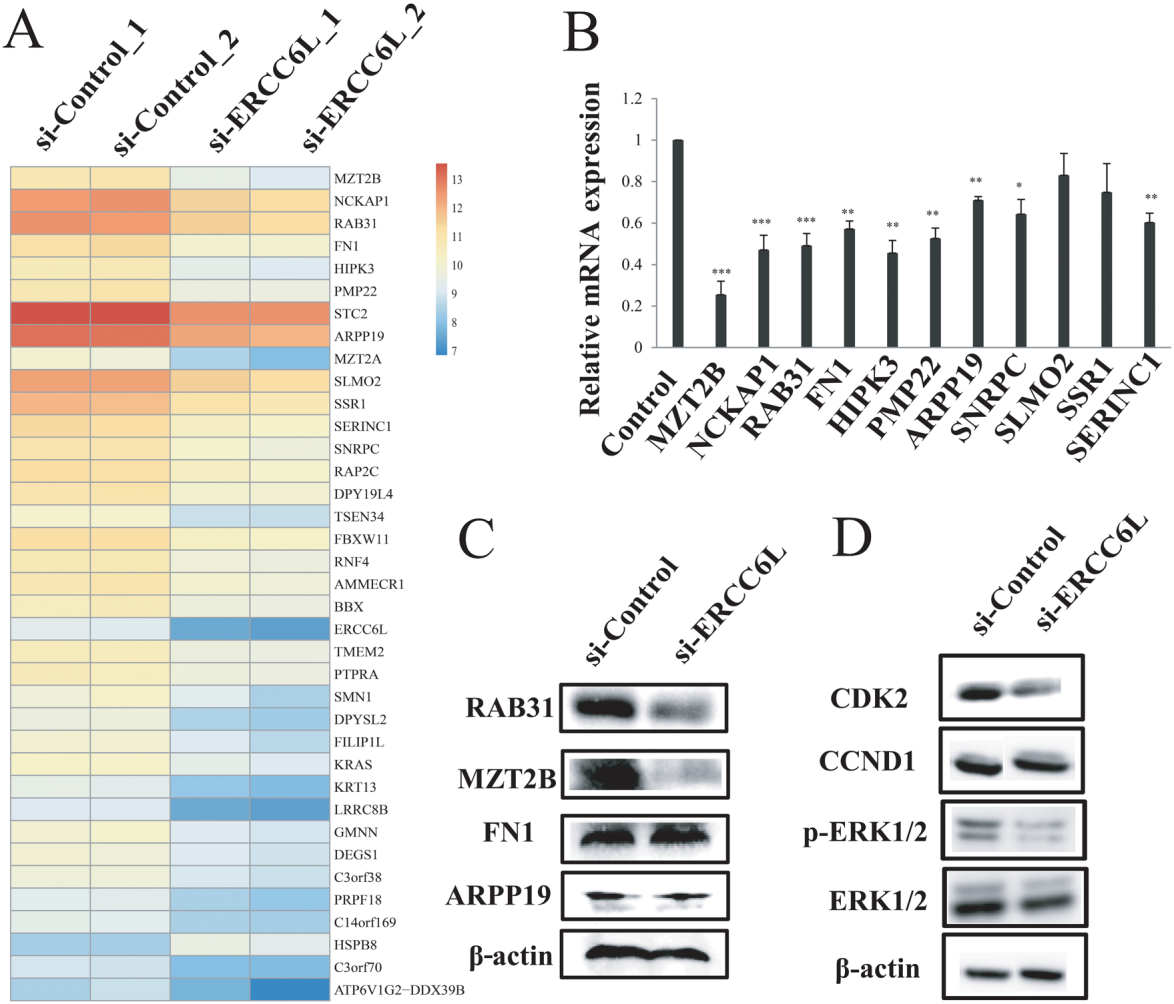
RNA-seq analysis after *ERCC6L* knockdown **A**. Heatmap of genes with differential expression in MCF-7 cells after transfection. **B**. Validation of differentially expressed genes (DEGs) by qRT-PCR. β-actin was used as the internal control. **C**. Validation of reported cancer-associated DEGs (RAB31, MZT2B, FN1, and ARPP19) by western blot. β-actin was used as the internal control. **D**. Mitogen-activated protein kinase (MAPK), cyclin dependent kinase 2 (CDK2) and cyclin D1 (CCND1) protein levels after *ERCC6L* knockdown in MCF-7 cells. *p<0.05 compared to the si-control group. **p<0.01 compared to the si-control group. ***p<0.001 compared to the control group.

To explore the mechanism of *ERCC6L* silencing in inhibiting cancer cell proliferation, we chose MCF-7 cell line to perform RNA-seq analysis after *ERCC6L* knockdown. We identified 77 significantly differentially expressed genes (DEGs) (e.g. *MZT2B*, *NCKAP1*, *RAB31*, *FN1*, *HIPK3*, *PMP22*, *STC2*, and *ARPP19*) between si-ERCC6L and si-Control groups (Figure 3A and Supplementary Table 1). Gene ontology (GO) analysis showed that the DEGs were mainly enriched in several important biological processes, such as cell death (*p* = 2.28E-05), death (*p* = 2.68E-05) and regulation of cytoskeleton organization (*p* = 9.20E-04) (Supplementary Figure 2). To confirm the RNA-seq result, we determined the mRNA levels of top 11 DEGs (*MZT2B*, *NCKAP1*, *RAB31*, *FN1*, *HIPK3*, *PMP22*, *STC2*, *ARPP19*, *SLMO2*, *SSR1*, and *SERINC1*) by quantitative real-time PCR (qRT-PCR). As a result, the *MZT2B*, *NCKAP1*, *RAB31*, *FN1*, *HIPK3*, *PMP22*, *STC2*, *ARPP19* and *SERINC1* were markedly downregulated after *ERCC6L* silencing (Figure 3B). Of them, *RAB31*, *MZT2B*, *FN1*, and *ARPP19* have been reported to be associated with tumorigenesis (Supplementary Table 2) and were thus chosen for further protein validation. Finally, *RAB31* and *MZT2B* were found to be downregulated by *ERCC6L* silencing at the translational level (Figure 3C), while the *FN1* and *ARPP19* were not changed. *RAB31* was an upstream protein in the MAPK pathway and was involved in regulating cell proliferation [10]. *ERCC6L* silencing decreased the phosphorylation level of MAPK in breast cancer cells (Figure 3D and Supplementary Figure 3). CDK2 and Cyclin D1 (CCND1) are two proteins in the regulation of cell cycle from G1 to S phase [11, 12], which are two downstream target proteins of MAPK. We found that *ERCC6L* silencing did not change CCND1 expressions no matter at the transcriptional or translational levels (Figure 3D and Supplementary Figure 4). However, *ERCC6L* silencing can reduce CDK2 protein expression (Figure 3D).

### *ERCC6L* silencing inhibited tumor growth in nude mice

**Figure 4:**
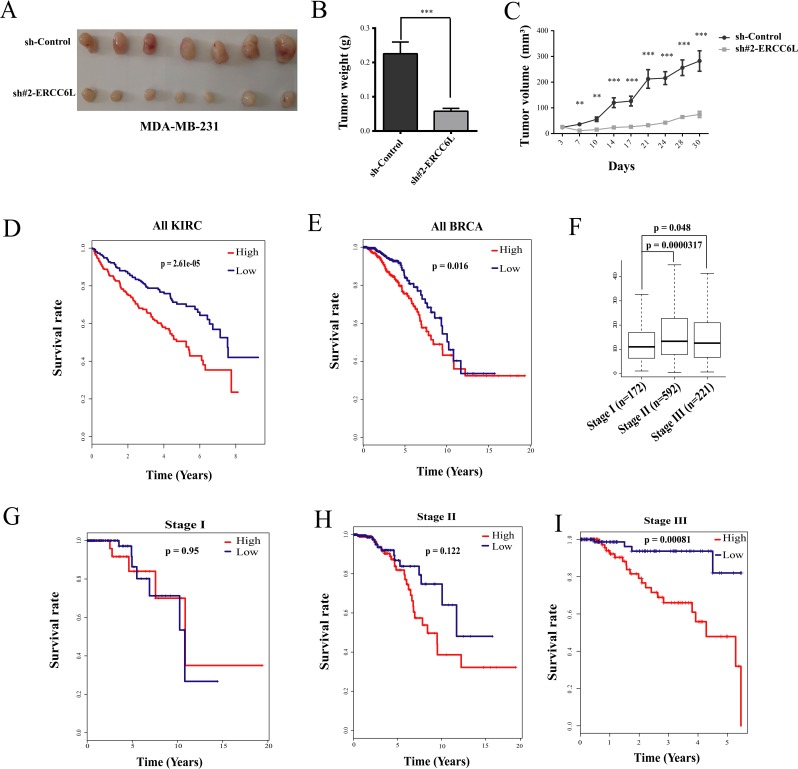
Effect of *ERCC6L* silencing on tumor growth and associations of *ERCC6L* expression with clinical survival and progress of breast and kidney cancer **A**. Tumor masses were harvested from MDA-MB-231-sh-Control and MDA-MB-231-sh#2 *ERCC6L* after tumors had grown for one month. **B**. Tumor weight after knockdown of *ERCC6L*. **C**. Changes in tumor volume after knockdown of *ERCC6L* in nude mice. **D**. Kaplan-Meier survival analysis in KIRC from TCGA. **E**. Kaplan-Meier survival analysis in BRCA from TCGA. **F**. Expression of *ERCC6L* at stage I, stage II, and stage III in breast cancer. **G**. Association of *ERCC6L* expression with survival at stage I in breast cancer. **H**. Association of *ERCC6L* expression with survival at stage II in breast cancer. **I**. Association of *ERCC6L* expression with survival at stage III in breast cancer. The clinical samples were devided into two groups according *ERCC6L* mRNA expression levels. High expression indicates that the mRNA value is greater than the median value of all tumors samples; Low expression indicates that the mRNA value is lower than the median value of all tumors samples. *p<0.05 compared to the sh-control group. **p<0.01 compared to the sh-control group. ***p<0.001 compared to the sh-control group.

To verify the role of *ERCC6L* in *in-vitro* experiment, we chose the invasive breast cancer cell line (MDA-MB-231) to perform xenograft tumor experiment in nude mice. The result showed that knockdown of *ERCC6L* strikingly suppressed the xenograft growth after 7 days of xenograft (Figure 4A-4C). However, body weight of mice was not significantly affected (Supplementary Figure 5).

### Associations of clinical survival with *ERCC6L* expression in breast and kidney cancer tumors

Since *ERCC6L* knockdown can inhibit cancer cell proliferation *in vitro* and tumor growth *in vivo*, we wonder whether it was associated with clinical survivals. As shown in Figure 4D and 4E, both kidney and breast cancer patients with a higher expression level of *ERCC6L* showed worse outcomes. We further analyzed the association of *ERCC6L* expression with survival rates at different stages in breast and kidney cancers. *ERCC6L* was obviously overexpressed in patients at stage II and III phase compared to those at stage I in breast cancer (Figure 4F). Moreover, high expression of *ERCC6L* showed gradually worse outcome in breast cancer (Figure 4G-4I), showing significant difference at stage IV in breast cancer (Figure 4I) and in kidney cancer (Supplementary Figure 6), suggesting that the high expression of *ERCC6L* is an effective prognostic biomarker in cancers, at least in breast and kidney cancers.

## DISCUSSION

In this study, we found that *ERCC6L* gene was consitently overexpressed in 12 solid tumors. It has been reported that *ERCC6L* combines with mitosis regulation kinase *PLK1* and responds to remodel centromeric chromatin. As a coactor of *ERCC6L*, *PLK1* has been well known as a genetic marker of tumor cell proliferation [13–15]. What is known about *ERCC6L* is its role in maintaining cell chromosome instability [6] and embryonic development [8], suggesting that *ERCC6L* might be a potential oncogene. Here we report for the first time that *ERCC6L* knockdown significantly inhibited cell proliferation *in vitro* and supressed tumor growth *in vivo*, and its high expression is significantly associated with bad outcome and progress in cancers, at least in breast and kidney cancers.

Cell cycle and apoptosis are two main performance in cancer cells [16]. We showed that apoptosis was not changed by *ERCC6L* knockdown, but cell cycle was significantly arrested at G0/G1 phase. These effects were validated in two breast and one kideny cancer cell lines. Cell cycle arrest is activated by DNA damage and/or malfunction of other critical organelles or structures (e.g. faulty mitotic spindle), which may cause apoptotic cascade [17]. However, it seems that the cell cycle arrest induced by *ERCC6L* knockdown was not enough to trigger cell apoptosis. This was supported by the unchanged protein expression of Cyclin D1 (CCND1), a key protein linked cell cycle to apoptosis [18]. Nevertheless, we can not exlude the possibility that *ERCC6L* silencing may induce other types of cell death, e.g. autophagy.

Our RNA-seq analysis identified the *RAB31* as a candidate target of *ERCC6L* as it was the most down-regulated gene at both the transcriptional and translational levels after *ERCC6L* silencing. Interestingly, *RAB31* gene is member of the Ras superfamily, which can promote cell proliferation and migration in breast cancer cells [19]. Moreover, *RAB31* has been reported to be an upstream regulator for MAPK [10]. ERK has been proved to be the best characterized MAPK and is considered to be a major regulator for cell growth and proliferation [20–23]. We found that the phosphorylated MAPK level was significantly suppressed by *ERCC6L* gene silencing, suggesting MAPK to be a likely downstream target of *ERCC6L*. Further, CDK2 and CCND1 are two proteins in the regulation of cell cycle from G1 to S phase [11, 12], acting as the downstream proteins of ERK [24, 25]. Although CCND1 expression was not changed by *ERCC6L* silencing in this study, CDK2 was markedly inhibited by *ERCC6L* knockdown (Figure 3D). These findings reveal that *ERCC6L* may reduce MAPK and CDK2 expressions through downregulating *RAB31*, thereby inhibiting cancer cell proliferation. These effects of *ERCC6L in vitro* were validated by its inhibitory role in tumor growth *in vivo*, the latter exerts more obvious effect than the former.

The survival rate is an important indicator of clinical prognosis. We observed that the *ERCC6L* mRNA increased along with the progress of breast cancer and associated with poor overall survival, at least in breast and kidney cancers, suggesting it to be an effective marker for cancer progression.

In conclusion, in this study, we report for the first time that *ERCC6L* may stimulates cancer cell proliferation and promotes tumor growth. Beasue *ERCC6L* is consistently and highly expressed in most solid cancers, our findings imply that it could be a good potential biomarker and provide new insight into the role of *ERCC6L* in oncogenesis of solid tumors.

## MATERIALS AND METHODS

### Data acquisition

Gene expression data of 4987 samples (4415 tumors and 572 matched non-cancerous tissues) from 12 cancer types (Supplementary Table 3), and clinical data for breast and kidney cancers were downloaded from The Cancer Genome Atlas (TCGA, http://cancergenome.nih.gov, RNA-Seq Version 2). In our study, the gene expression data sets (level 3) have been processed by a slight normalization method in which the values of each individual sample were divided by 75 percentile to compare gene expression levels among samples in TCGA.

### Cell culture and siRNA/shRNA transfection

Cell lines (MCF-7, MDA-MB-231 and 786-0) were bought from Conservation Genetics CAS Kunming Cell Bank and MCF-7 cells were cultured in DMEM/HIGH Glucose (HyClone, Logan, UT, USA), MDA-MB-231 were maintained in DMEM/F-12 (HyClone, Logan, UT, USA), 786-0 were cultured in RPMI medium (HyClone, Logan, UT, USA), supplemented with 10% fetal bovine serum (FBS) (Corning, Corning, NY, USA) and 1% penicillin/streptomycin (Gibco, Grand Island, NY, USA). MCF-7 cells were transfected with si-ERCC6L (target sequence: 5′-GCUGGUUAAUGACGUCUAA-3′) and control siRNA using riboFECT™ CP Transfection Kit (Ribobio, Guangzhou, Guangdong, China) according to the manufacturer's instructions. Simply, 20 μm siRNA was added to 1X riboFECT™ CP Buffer for 5 minutes and mixed with riboFECT™ CP Reagent for 15 minutes, then was added to medium at a final concentration of 50 nm. The siRNA were bought from Ribobio.

For generation of shRNA stable cell population, independent shRNAs against *ERCC6L* targeting to different regions (sh#1, target sequence: 5′-ACAAGATCTCTCCAGTATAAA-3′; sh#2, target sequence: 5′-CCTGGCTAAGAGAACCTGTAT-3′) were constructed using pLKO.1 vector. Supernatants containing different lenti-viruses generated from HEK-293T cells were collected. Then MDA-MB-231 and 786-0 cells were infected by lentiviruses and selected using puromycin for 3 passages. Quantitative real-time PCR (qRT-PCR) was used to determine the effectiveness of the siRNA and shRNA knockdown.

### Proliferation assay

MCF-7 cells were seeded at a density of 3 × 10^3^ cells in 96-well plates. After 12 h, MCF-7 cells were transfected with si-ERCC6L and si-Control. Cell viability was determined using CellTiter 96® AQueous One Solution Cell Proliferation Assay (Promega, Madison, WI, USA) on each day according to the manufacturer's protocol. After incubation for 3 h at 37°C, the absorbance was measured at 490 nm on a plate reader (Synergy H1, BioTek, Winooski, VT, USA).

MDA-MB-231 and 786-0 cells were seeded at a density of 3 × 10^3^ cells in 96-well plates. After 12 h, cell viability was also determined using the CellTiter 96® AQueous One Solution Cell Proliferation Assay kit according to the manufacturer's protocol.

### Quantitative RT-PCR (qRT-PCR)

Cells were collected at 48 h after transfection. Total RNA were extracted using Trizol reagent (Invitrogen, Carlsbad, CA, USA). Reverse transcription was performed with random nucleotide primers using GoScript™ reverse transcription system according to the manufacture's protocol (Promega, Madison, WI, USA). qRT-PCR with gene-specific primers was performed using GoTaq qPCR master mix (Promega, Madison, WI, USA). β-actin was used as the internal control. The expression of target genes was calculated by 2^−(Δ-ΔCT)^ method. The primers were shown in Supplementary Table 4.

### Western blot

Cells were lysed with RIPA Lysis Buffer (Beyotime, Haimen, Jiangsu, China). Proteins were quantified by BCA Protein Assay Kit (Beyotime, Haimen, Jiangsu, China). 20-50 μg of total proteins were loaded onto SDS-PAGE and subsequently transferred to PVDF membranes (Bio-Rad, Richmond, CA, USA). Membranes were incubated with the following primary antibodies: anti-RAB31, anti-FN1, anti-MZT2B, anti-ARPP19, anti-ERK1/2 and anti-p-ERK1/2 (Santa Cruz Biotechnology, Santa Cruz, CA, USA), and anti-β-actin (Beyotime, Haimen, Jiangsu, China). The detailed protocol was described in our previous study [26].

### Apoptosis assay and cell cycle analysis

Cells were harvested at 48 h after transfection, apoptosis were determined using the Annexin V apoptosis detection kit (eBioscience, San Diego, CA, USA) according to manufacturer's protocol. Cells were collected 48 h after transfection and fixed with cold 75% alcohol at 4°C overnight. Alcohol was removed and cells were washed with cold phosphate buffer saline (PBS). Cells were stained with propidium iodide solution containing 20 μg/ml RNase and incubated at room temperature for 30 min. After filtration by a nylon mesh filter, cell cycle was performed on a fluorescence-activated cell sorting (FACS, FACSVerse) analysis. Data were analyzed using the Flowjo software (Version 7.6.1, Tree Star Software, San Carlos, CA, USA).

### Tumorigenesis in nude mice

For the MDA-MB-231 xenograft tumor growth experiment, a total of 10 female BALB/c mice at 5 weeks of age (Vital River, Beijing) were randomly divided into 2 groups. MDA-MB-231-sh-Control and MDA-MB-231-sh#2 ERCC6L cells (4 × 10^6^ cells/point subcutaneously) were injected into inguen at two sides of each mouse. Tumor sizes in two groups were measured twice per week for one month using vernier calipers once tumors became palpable. Tumor volume was calculated using the following equation: tumor volume (mm^3^) = (length × width^2^)/2. All mice were sacrificed at the end of the experiment and tumors were harvested and weighed.

### RNA extraction and RNA sequencing

After *ERCC6L* silencing, total RNA was isolated using Trizol reagent according to the manufacturer's instructions. Poly(A) mRNA was isolated using oligo (dT) beads from 1 μg of total RNA for each sample. The construction of RNA-seq library was based on TruSeqTM RNA library preparation protocol. RNA-seq was completed by Novogene company (Bejing, China). The raw data of RNA-seq were processed using CASAVA V1.6 package. The quality control of each sample was accomplished using FASTQC V2_1.2.10. Clean reads were used in the whole analysis process.

### Gene mapping and gene annotation

The human genomic data hg 19 build and gene annotation information was downloaded from UCSC database (http://genome.ucsc.edu/). We mapped the clean reads onto human genome hg 19 build using the alignment software Bowtie2 (https://sourceforge.net/projects/bowtie-bio/files/bowtie2/2.2.5/). Then we obtained transcript quantification from RNA-Seq data and calculated gene expression level using RSEM V1.1.17 software (http://deweylab.github.io/RSEM/). To estimate the level of gene expression, the reads count was transformed into reads per kilo bases per million mapped reads (RPKM) which reflects the abundance of gene expression [27]. The values of each individual sample were divided by 75 percentile to compare gene expression levels among samples. We identified differentially expressed genes using NOISeq package [28]. The Generic Annotation File (GAF) that provides all of our annotations for genes was downloaded from TCGA database (https://tcga-data.nci.nih.gov/tcgafiles).

### Gene set enrichment analysis

Gene ontology biological process and pathway were analyzed using the David database (https://david.ncifcrf.gov/). Gene list for biological process and pathway was got from GeneCards (http://www.genecards.org).

### Statistical analysis

Statistical analyses were performed using R software (V 2.6) (http://www.R-project.org/). *P* values less than 0.05 were considered statistically significant. In RNA-seq analysis, probability of gene expression difference > 0.8 is considered differentially expressed.

## SUPPLEMENTARY MATERIALS FIGURES AND TABLES




