# Supplementary material for: ERCC6L, a DNA helicase, is involved in cell proliferation and associated with survival and progress in breast and kidney cancers
Source: Oncotarget. 2017 Feb 2;8(26):42116–24. doi: 10.18632/oncotarget.14998 (PMC5522053; doi:10.18632/oncotarget.14998)
Supplement: Supplementary file 1 [file oncotarget-08-42116-s001.pdf]

ERCC6L, a DNA helicase, is involved in cell proliferation and associated with survival and progress in breast and kidney cancers

SUPPLEMENTARY MATERIALS

REFERENCES

1. Grismayer B, Solch S, Seubert B, Kirchner T, Schafer S, Baretton G, Schmitt M, Luther T, Kruger A, Kotzsch M, Magdolen V. Rab31 expression levels modulate tumor-relevant characteristics of breast cancer cells. Mol Cancer. 2012; 11:62.
2. Pan Y, Zhang Y, Chen L, Liu Y, Feng Y, Yan J. The critical role of Rab31 in cell proliferation and apoptosis in cancer progression. Mol Neurobiol. 2015; 7:4431-4437.
3. Li W, Pung D, Su ZY, Guo Y, Zhang C, Yang AY, Zheng X, Du ZY, Zhang K, Kong AN. Epigenetics reactivation of Nrf2 in prostate TRAMP C1 cells by curcumin analogue FN1. Chem Res Toxicol. 2016; 29:694-703.
4. Song HY, Pan JL, Liu Y, Wen HZ, Wang L, Cui JF, Liu YK, Hu B, Yao ZM, Ji G. Increased ARPP-19 expression is associated with hepatocellular carcinoma. 2015; 16:178-192.
5. Choi YK, Liu P, Sze SK, Dai C, Qi RZ. CDK5RAP2 stimulates microtubule nucleation by the gamma-tubulin ring complex. J Cell Biol. 2010; 191:1089-1095.
6. Suzuki T, Nishiyama K, Yamamoto A, Inazawa J, Iwaki T, Yamada T, Kanazawa I, Sakaki Y. Molecular cloning of a novel apoptosis-related gene, human Nap1 (NCKAP1), and its possible relation to Alzheimer disease. Genomics. 2000; 63:246-254.
7. Curtin JF, Cotter TG. JNK regulates HIPK3 expression and promotes resistance to Fas-mediated apoptosis in DU 145 prostate carcinoma cells. J Biol Chem. 2004; 279:17090-17100.
8. Liu S, Chen Z. The functional role of PMP22 gene in the proliferation and invasion of osteosarcoma. Med Sci Monit. 2015; 21:1976-1982.
9. Will CL, Rumpler S, Gunnewiek JK, vanVenrooij WJ, Luhrmann R. *In vitro* reconstitution of mammalian U1 snRNPs active in splicing: the U1-C protein enhances the formation of early (E) spliceosomal complexes. Nucleic Acids Res. 1996; 24:4614-4623.
10. Hendrickson SL, Lautenberger JA, Chinn LW, Malasky M, Sezgin E, Kingsley LA, Goedert JJ, Kirk GD, Gomperts ED, Buchbinder SP, Troyer JL, O'Brien SJ. Genetic variants in nuclear-encoded mitochondrial genes influence AIDS progression. PloS One. 2010; 5:e12862.
11. Mesbah K, Camus A, Babinet C, Barra J. Mutation in the Trap alpha/Ssr1 gene, encoding translocon-associated protein alpha, results in outflow tract morphogenetic defects. Mol Cell Biol. 2006; 26:7760-7771.
12. Ren WH, Yang CY, Yang XM, Yu L. siRNA- mediated knockdown of hTDE2 retards cell cycle progression through transcriptional activation of p21. Oncol Rep. 2014; 31:1314-1322.
13. Mortazavi A, Williams BA, McCue K, Schaeffer L, Wold B. Mapping and quantifying mammalian transcriptomes by RNA-Seq. Nat Methods. 2008; 5:621-628.

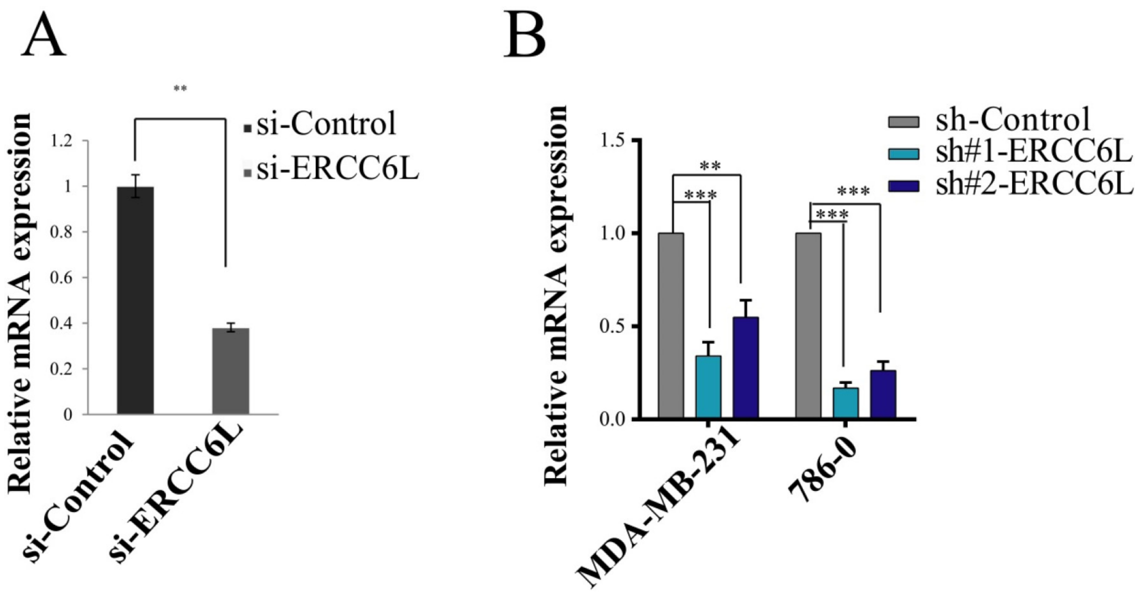

Supplementary Figure 1: Efficiency of ERCC6L knockdown in cancer cells. A. Efficiency of ERCC6L knockdown using siRNA in MCF-7 cell lines. **B.** Efficiency of ERCC6L knockdown using shRNA in MDA-MB-231 and 786-0 cells. ERCC6L was knocked down by two shRNAs (sh#1-ERCC6L and sh#2-ERCC6L) in both cell lines. *p<0.05 compared to the control group. **p<0.01 compared to the control group. ***p<0.001 compared to the control group.

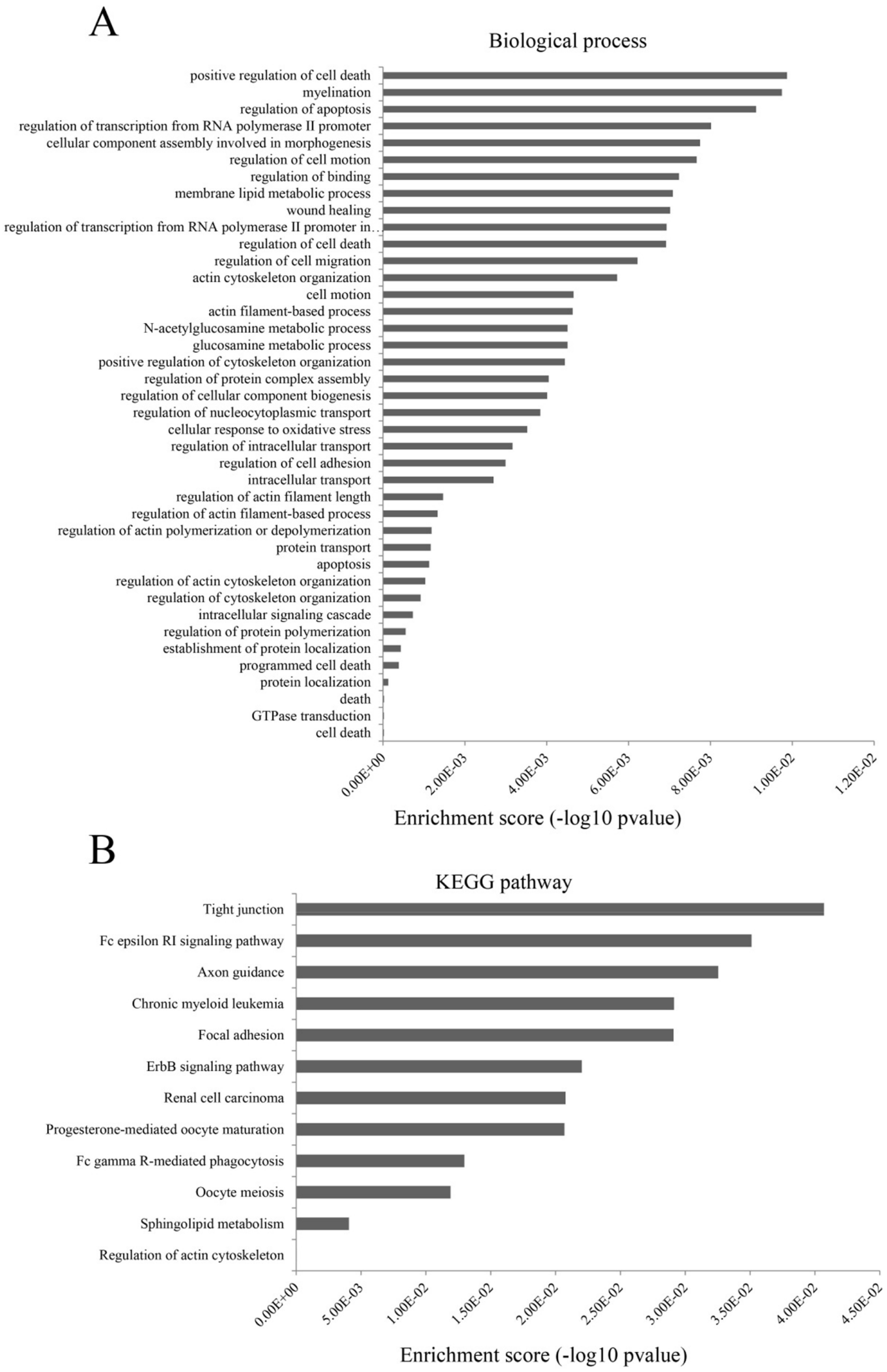

Supplementary Figure 2: Gene Ontology (GO) enrichment analysis on differently expressed genes (DEGs). A. Biological process enrichment analysis. **B.** KEGG pathway enrichment analysis.

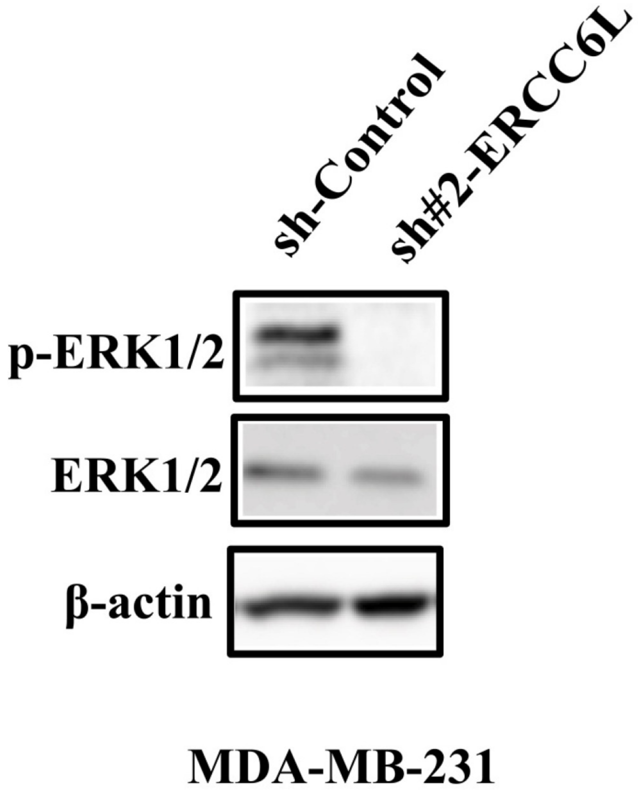

Supplementary Figure 3: Effect of ERCC6L silencing on MAPK protein levels in MDA-MB-231 cells.

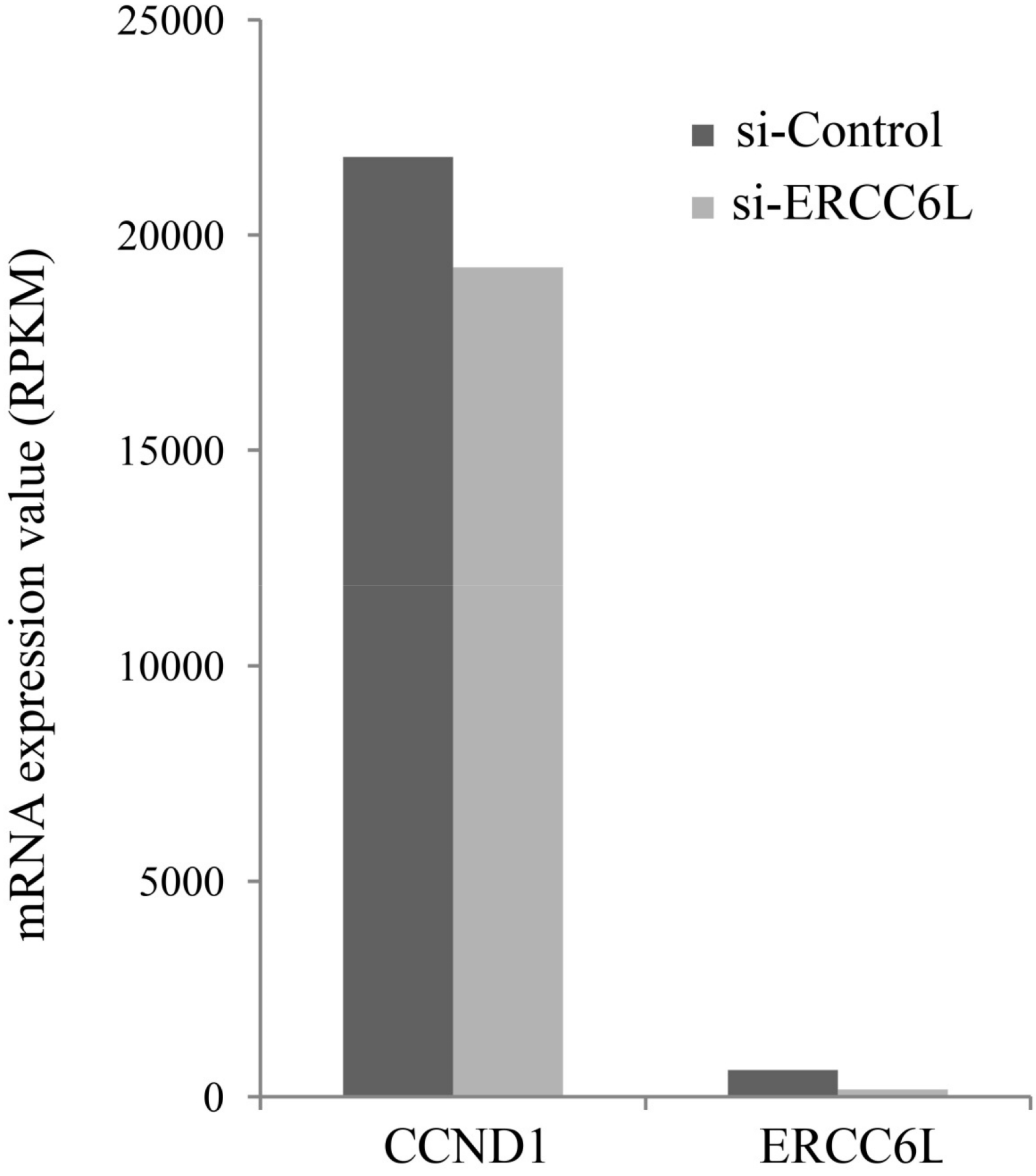

Supplementary Figure 4: mRNA level of cyclin D1 (CCND1) after *ERCC6L* silencing in MCF7 cells. Ordinate dimension indicates reads per kilo bases per million mapped reads (RPKM) value [13] which reflects the abundance of a gene expression.

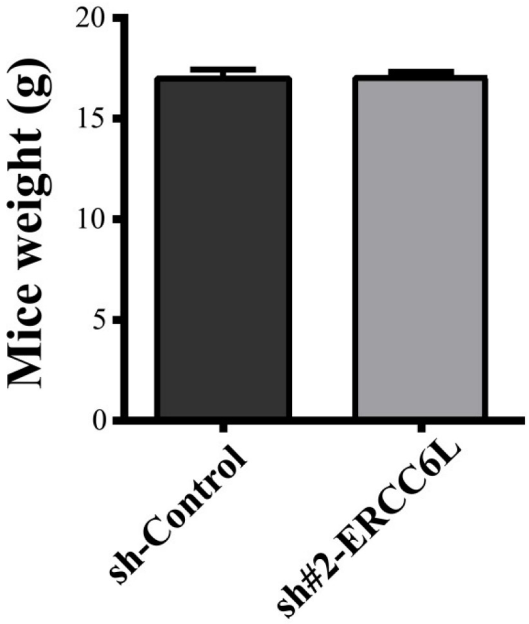

Supplementary Figure 5: Body weight of mice undergone xenograft. No difference in body weight was oberserved between the sh-Control and sh-ERCC6L groups.

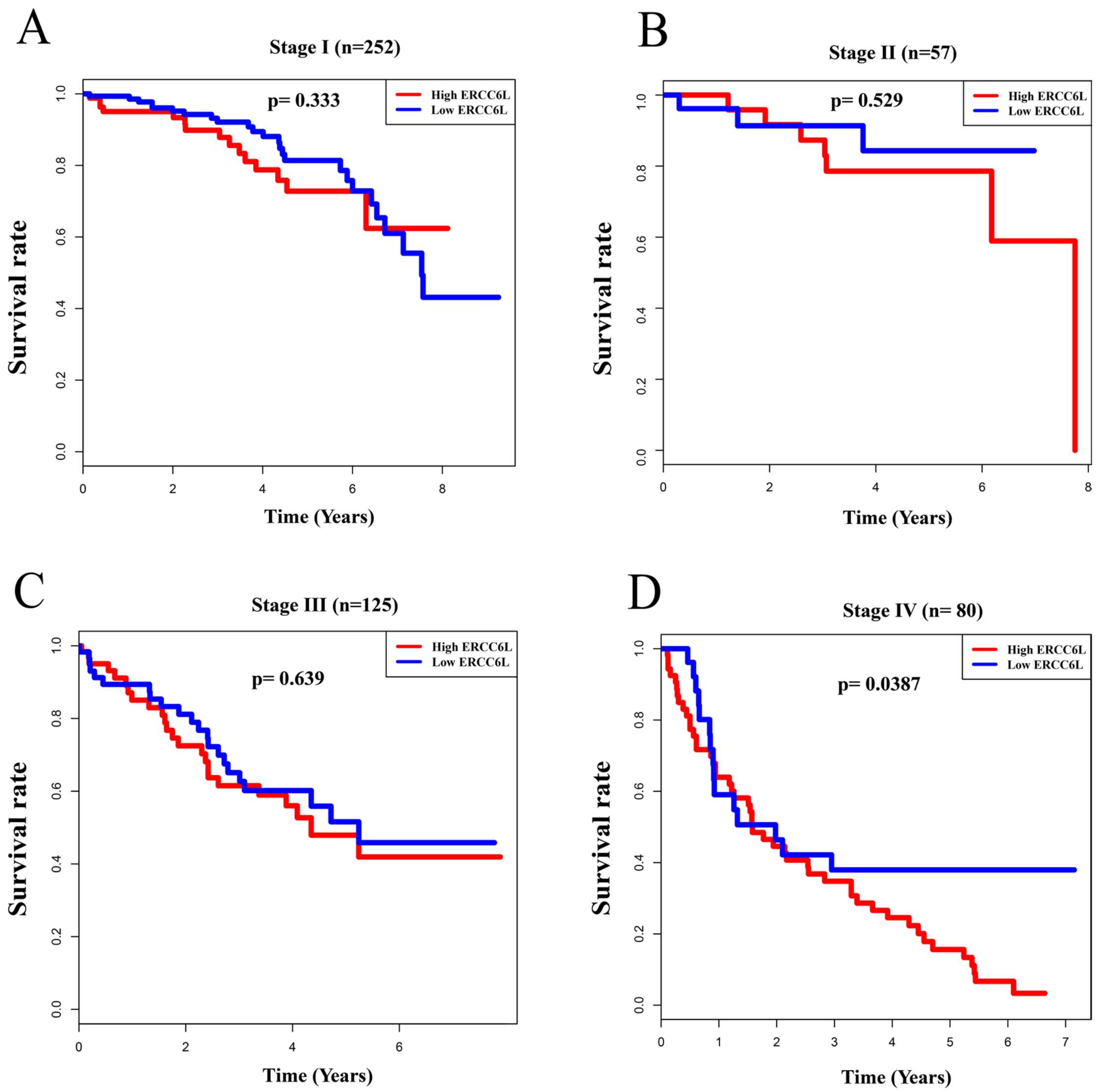

Supplementary Figure 6: Kaplan-Meier survival analysis at different stages of KIRC from The Cancer Genome Atlas (TCGA) database. The clinical samples were devided into two groups according to ERCC6L mRNA expression levels. High expression indicates that the mRNA value is greater than the median value of all tumors samples; Low expression indicates that the mRNA value is lower than the median value of all tumors samples. **A.** Association of ERCC6L expression with clinical survival at stage I in kidney cancer. **B.** Association of ERCC6L expression with clinical survival at stage II in kidney cancer. **C.** Association of ERCC6L expression with clinical survival at stage III in kidney cancer. **D.** Association of ERCC6L expression with clinical survival at stage IV in kidney cancer.

Supplementary Table 1: List of differently expressed genes (DEGs)

See Supplementary File 1

Supplementary Table 2: Functional description of top 11 differently expressed genes (DEGs)

Symbol	Description	References
RAB31	*RAB31* can promote cell proliferation and migration in breast cancer cells; *RAB31* promotes cell growth and migration and induces the expression of EMT.	[1, 2]
FN1	Epigenetics reactivation of Nrf2 in prostate TRAMP C1 cells by curcumin analogue FN1.	[3]
ARPP19	Increased ARPP-19 expression is associated with hepatocellular carcinoma.	[4]
MZT2B	Mitotic spindle organizing protein 2B. It affects cell cycle.	[5]
NCKAP1	NCKAP1 could be related to Alzheimer disease.	[6]
HIPK3	There is only a report that JNK regulates HIPK3 expression and promotes resistance to Fas-mediated apoptosis in DU 145 prostate carcinoma cells.	[7]
PMP22	PMP22 mainly expressed in peripheral nervous. There is a report that PMP22 affect cell proliferation and invasion.	[8]
SNRPC	SNRPC enhances the formation of early spliceosomal complexes. There is not tumor-related report.	[9]
SLMO2	SLMO2 affect AIDS progression.	[10]
SSR1	SSR1 is associated with endoplasmic reticulum protein transposition. There is not tumor-related report.	[11]
SERINC1	There is only a report that knocked down the expression of TDE2 caused cell cycle arrest at G2.	[12]

Supplementary Table 3: Numbers and types of normal and corresponding cancer tissues from The Cancer Genome Atlas (TCGA) databases

Name	Abbreviation	Number of cancer	Number of normal
Bladder urothelial carcinoma	BLCA	241	19
Breast invasive carcinoma	BRCA	1037	110
Colon adenocarcinoma	COAD	261	41
Head and neck squamous cell carcinoma	HNSC	497	43
Kidney chromophobe	KICH	66	25
Kidney renal clear cell carcinoma	KIRC	518	72
Kidney renal papillary cell carcinoma	KIRP	172	30
Liver hepatocellular carcinoma	LIHC	191	50
Lung adenocarcinoma	LUAD	488	58
Lung squamous cell carcinoma	LUSC	489	50
Prostate adenocarcinoma	PRAD	297	50
Uterine corpus endometrial carcinoma	UCEC	158	24
Total		4415	572

Supplementary Table 4: Sequence of primers

Primer	Sequences (5′ to 3′)
MZT2B-F	ACCCGAGGGAGAAACAAAGG
MZT2B-R	TGCTGGGGACAAAGATGCAA
NCKAP1-F	TCATGGTGTTTGTGGCAGTT
NCKAP1-R	AAAGCTGCAGCAATCTGGTT
RAB31-F	CTGGTCAGGAACGGTTTCAT
RAB31-R	ATGGCACCTATGGATTCAGC
FN1-F	GACGCATCACTTGCACTTCT
FN1-R	GCAGGTTTCCTCGATTATCCT
HIPK3-F	CTGAGAGTGTGGCTGGTTCA
HIPK3-R	CCAGACAACATGTGCAATCC
PMP22-F	CCTGTCGATCATCTTCAGCA
PMP22-R	AGCACTCATCACGCACAGAC
ARPP19-F	TCCTCATCTGGGACAAAAGC
ARPP19-R	TTCCGTTGAGGAAGGTCTTG
SNRPC-F	CCTCACCCATGACTCTCCAT
SNRPC-R	CAGGAGGAGGAGCAGAGAAT
SLMO2-F	AAGATCTGGACTTCGGAGCA
SLMO2-R	TCTGTGGCTGTGCAACTTTC
SSR1-F	TGCCTCATTCCGTTATCCTC
SSR1-R	TTGGAATACATTGCCGTTCA
SERINC1-F	GCAGGTGCCTTTTGTTTCAT
SERINC1-R	TGAACAACTGGCTGGATGAG
