# Supplementary material for: ERCC6L, a DNA helicase, is involved in cell proliferation and associated with survival and progress in breast and kidney cancers
Source: Oncotarget. 2017 Feb 2;8(26):42116–24. doi: 10.18632/oncotarget.14998 (PMC5522053; doi:10.18632/oncotarget.14998)
Supplement: Supplementary file 2 [file oncotarget-08-42116-s002.docx]

**Supplementary Materials**

**Supplemental Table 1. List of differently expressed genes (DEGs).**

| **Symbol** | **log2Ratio(E4/ENC)** | **Probability** |
| --- | --- | --- |
| MZT2B | -1.730788663 | 0.943068243 |
| NCKAP1 | -1.315570492 | 0.935706609 |
| RAB31 | -1.249044361 | 0.930816765 |
| FN1 | -1.380375949 | 0.929070392 |
| HIPK3 | -1.557063783 | 0.926518001 |
| PMP22 | -1.412801428 | 0.925604514 |
| STC2 | -1.051335944 | 0.923186459 |
| ARPP19 | -1.056958006 | 0.922756582 |
| MZT2A | -1.812487374 | 0.922031166 |
| SLMO2 | -1.030653852 | 0.918699624 |
| SSR1 | -1.044430085 | 0.916577109 |
| SERINC1 | -1.121529288 | 0.914159054 |
| SNRPC | -1.158612416 | 0.911364858 |
| RAP2C | -1.045331759 | 0.909188608 |
| DPY19L4 | -1.112727938 | 0.908812466 |
| TSEN34 | -1.454039828 | 0.906340677 |
| FBXW11 | -1.001267774 | 0.906206341 |
| RNF4 | -1.126582692 | 0.904755508 |
| AMMECR1 | -1.065998875 | 0.904110693 |
| BBX | -1.037163781 | 0.895029554 |
| ERCC6L | -1.925352131 | 0.894787749 |
| TMEM2 | -1.032347129 | 0.893417517 |
| PTPRA | -1.015524725 | 0.892987641 |
| SMN1 | -1.258534954 | 0.89220849 |
| DPYSL2 | -1.464702787 | 0.892047286 |
| UNKL | 0.99617406 | 0.886378291 |
| FILIP1L | -1.20649727 | 0.883610962 |
| KRAS | -1.019378688 | 0.877888232 |
| KRT13 | -1.481031345 | 0.875362708 |
| LRRC8B | -1.725732162 | 0.875335841 |
| GMNN | -1.06497266 | 0.874153681 |
| DEGS1 | -1.031949913 | 0.863997851 |
| C3orf38 | -1.031034744 | 0.861821601 |
| PRPF18 | -1.167471576 | 0.84486835 |
| LMNB1 | -0.951753956 | 0.835787211 |
| C14orf169 | -1.069431659 | 0.834282644 |
| SLC7A2 | -0.899911609 | 0.833933369 |
| ARF4 | -0.949287842 | 0.831810854 |
| PTP4A2 | -0.915540741 | 0.83057496 |
| HSPB8 | 1.018593771 | 0.829365932 |
| PITRM1 | -0.984530543 | 0.829150994 |
| DCTN5 | -0.955070028 | 0.828909189 |
| RTN4 | -0.885825233 | 0.827001612 |
| GANAB | 0.822116323 | 0.826571736 |
| SLC2A1 | -0.90575333 | 0.825470177 |
| UHMK1 | -0.873582886 | 0.824126814 |
| ELP2 | -0.825909895 | 0.824046212 |
| SPOPL | -0.907543176 | 0.823750672 |
| ZDHHC20 | -0.819491855 | 0.823347663 |
| TMEM64 | -0.768838797 | 0.821547555 |
| HIGD1A | -0.931436632 | 0.821144546 |
| PARD6B | -0.813535344 | 0.820096722 |
| GPRC5A | -0.877716868 | 0.819478775 |
| PURB | -0.84282998 | 0.818887695 |
| L1CAM | 0.853443092 | 0.817195056 |
| LYPLA1 | -0.905705749 | 0.816281569 |
| COX6C | -0.755256948 | 0.815744224 |
| PKIB | -0.800754308 | 0.815556153 |
| UBE2D2 | -0.819725694 | 0.814508329 |
| KIF5B | -0.735812838 | 0.814185922 |
| VPS26A | -0.985009487 | 0.813997851 |
| KIAA1191 | -0.836335218 | 0.813567974 |
| C3orf70 | -1.153137432 | 0.809484148 |
| ATP6V1G2-DDX39B | -1.391618947 | 0.809081139 |
| MSMO1 | -0.893756471 | 0.809027405 |
| PAPSS2 | -0.882282281 | 0.806555615 |
| PCNXL3 | 0.779055956 | 0.80454057 |
| COX5A | -0.868942959 | 0.804352499 |
| SETD8 | -0.884221732 | 0.803465879 |
| LAMTOR5 | -0.858862854 | 0.802901666 |
| XPO4 | -0.817474254 | 0.80236432 |
| ABCF2 | -0.852354154 | 0.801585169 |
| MAPRE1 | -0.718854583 | 0.801585169 |
| ELF1 | -0.697931493 | 0.801450833 |
| OTUD4 | -0.971978681 | 0.801235895 |
| XBP1 | -0.653454036 | 0.800456744 |
| QKI | -0.854986497 | 0.800188071 |
